# Gastric Electrical Stimulation for Refractory Gastroparesis: A Promising Treatment Modality for Symptom Control and Gastric Emptying

**DOI:** 10.7759/cureus.41630

**Published:** 2023-07-10

**Authors:** Shahzeb Saeed, Muhammad Kamran, Kanwal Bhagwani, Nehal Shaikh, Chukwuyem Ekhator, Mohammed Farahat, Ali M Abdelaziz, Abdullah Shehryar

**Affiliations:** 1 Internal Medicine, Army Medical College, Islamabad, PAK; 2 Internal Medicine, Mayo Hospital, Lahore, PAK; 3 Medicine, Chandka Medical College, Larkana, PAK; 4 Department of Medicine, Ghulam Muhammad Mahar Medical College, Sukkur, PAK; 5 Neuro-Oncology, New York Institute of Technology, College of Osteopathic Medicine, Old Westbury, USA; 6 Internal Medicine, Souad Kafafi Hospital, 6 October, EGY; 7 Department of Internal Medicine, Alexandria University Faculty of Medicine, Alexandria, EGY; 8 Internal Medicine, Allama Iqbal Medical College, Lahore, PAK

**Keywords:** efficacy, quality of life, delayed gastric emptying, gastric electrical stimulation (ges), gastroparesis

## Abstract

Gastroparesis is a disorder with few available treatments and delayed stomach emptying. Gastric electrical stimulation (GES) has shown promise in treating the signs and symptoms of gastroparesis as well as gastric emptying by stimulating the stomach with high-frequency electrical impulses. In this case, a 43-year-old lady with refractory gastroparesis had a GES device laparoscopically implanted. Even though GES seems promising, more study is necessary to improve patient choice, technique, and long-term results. Patients with refractory gastroparesis who have not responded to traditional therapy should be considered for GES, with treatment decisions being made individually depending on clinical presentation and patient preferences.

## Introduction

Gastroparesis is a condition characterized by delayed gastric emptying, leading to symptoms such as nausea, vomiting, early satiety, and abdominal discomfort. The etiology of gastroparesis is multifactorial, with diabetes, post-surgical complications, neurological disorders, and idiopathic causes being the most common [1]. Treatment options for gastroparesis are limited, and many patients fail to achieve adequate symptom control with conventional therapies, significantly impacting their quality of life.

Gastric electrical stimulation (GES) is a therapeutic option that has emerged in recent years for the management of refractory gastroparesis. The use of electrical stimulation to modulate gastrointestinal motility dates back to the 1970s, but its clinical application was limited due to the technical difficulties involved in implanting the devices [2]. However, technological advances have made the implantation of GES devices a safe and effective treatment option for patients with refractory gastroparesis.

GES works by delivering high-frequency electrical impulses to the stomach, promoting contractions, and facilitating gastric emptying. The electrical impulses are delivered via electrodes placed on the stomach wall, which are connected to a stimulator device implanted in the patient's abdomen. The stimulator device is programmed to deliver electrical impulses at a specific frequency and intensity, tailored to each patient's needs [3].

Several studies have shown the efficacy of GES in the treatment of gastroparesis, with significant improvements in symptom control, gastric emptying, and quality of life reported in many cases. However, the long-term efficacy and safety of GES remain to be established, and the procedure's high cost and invasiveness limit its widespread use [4,5].

In this case report, we present a patient with refractory gastroparesis who achieved significant improvement in symptoms following GES. We discuss the patient's clinical presentation, management, and outcomes following GES placement, highlighting the potential benefits of this treatment modality for patients with refractory gastroparesis.

## Case presentation

A 43-year-old female presented to our clinic with a long-standing history of refractory gastroparesis. Despite optimal medical management, she had a history of nausea, vomiting, early satiety, and abdominal pain. Her symptoms had significantly impacted her quality of life, and she could not maintain her weight and had lost approximately 10 pounds over the past year. The patient reported multiple hospital admissions for severe nausea and vomiting, which had not responded to standard antiemetic therapy.

**Figure 1:**
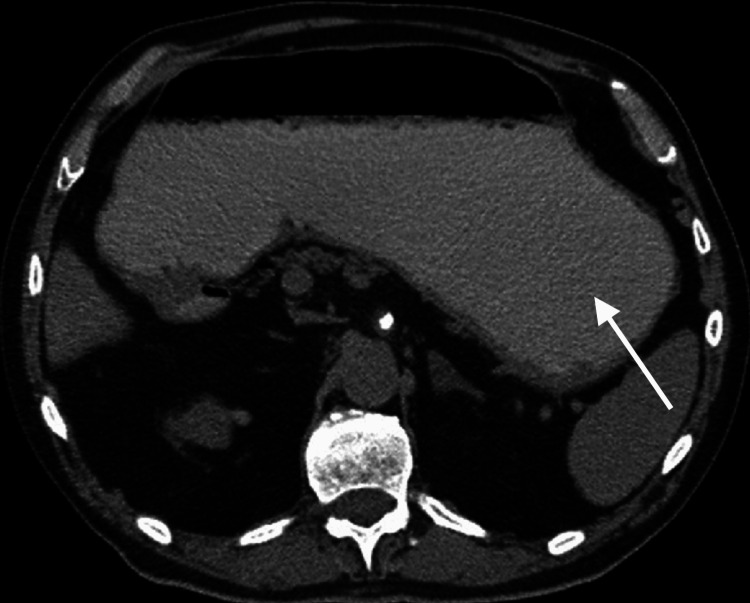
CT scan of abdomen indicating delayed gastric emptying.

The patient had undergone a diagnostic evaluation, including an esophagogastroduodenoscopy, an abdominal CT scan (Figure 1), and a gastric emptying study, which revealed delayed gastric emptying with a half-time of 146 minutes. She had previously failed multiple medical therapies, including prokinetic agents, antiemetics, and dietary modifications.

Given the patient's refractory symptoms, a multidisciplinary team, including gastroenterologists, surgeons, and nutritionists, was consulted to discuss alternative treatment options. GES was considered a potential therapy, and the patient was referred for further evaluation. The patient appeared pale and fatigued on physical examination, with mild epigastric tenderness. Laboratory tests revealed no abnormalities. A repeat gastric emptying study was performed, which confirmed delayed gastric emptying with a half-time of 140 minutes.

After thorough discussion and informed consent, the patient underwent laparoscopic placement of a GES system with two leads implanted in the gastric wall. The surgery was uneventful, and the patient was discharged home on postoperative day 2 with pain control and a clear liquid diet. The stimulation parameters were optimized over several weeks, and the patient was closely monitored for any adverse effects.

After GES placement, the patient reported a significant improvement in symptoms, with a decrease in nausea and vomiting, an improved appetite, and less frequent abdominal pain. She tolerated a regular diet and reported a weight gain of approximately 5 pounds over the next three months. A repeat gastric emptying study performed three months after the GES placement showed a significant improvement in gastric emptying, with a half-time of 80 minutes.

The patient reported significant symptom improvement at her follow-up visits, with no adverse effects from the GES therapy. She resumed her regular activities and reported a significant improvement in her quality of life.

## Discussion

Gastroparesis is a challenging condition to manage, with limited treatment options available for patients who fail to respond to conventional therapies. GES has emerged as a promising treatment modality for refractory gastroparesis, with several studies reporting improvements in symptom control, gastric emptying, and quality of life.

In this case report, we present a patient with refractory gastroparesis who achieved significant improvement in symptoms following the placement of a gastric electrical stimulator. The patient had a two-year history of severe nausea, vomiting, and early satiety, with little relief from medications and dietary modifications. The patient underwent the placement of a gastric electrical stimulator, and significant improvements in her symptoms were noted. The patient reported a decrease in nausea, vomiting, and early satiety, along with improved appetite and weight gain. The patient was able to tolerate a more varied diet, and her quality of life significantly improved.

Gastric emptying studies performed before and after GES placement showed a significant improvement in gastric emptying, with a decrease in the gastric retention percentage from 62% to 28% and 26%, respectively, at six- and twelve-month follow-ups. These findings are consistent with previous studies that have reported improvements in gastric emptying following GES placement.

In a recent multicenter randomized controlled trial, the effectiveness of GES in treating gastroparesis was evaluated. The study included 262 patients with refractory gastroparesis who were randomly assigned to receive either GES or sham therapy. The study showed that GES was associated with significant improvements in nausea, vomiting, abdominal pain, and overall symptom severity compared to sham therapy. The study also showed improvements in gastric emptying in patients who received GES [5].

The mechanism by which GES improves gastric motility has yet to be fully understood. It is thought that electrical stimulation of the stomach may promote the release of neurotransmitters, such as acetylcholine, which stimulate gastric motility. GES may also reduce the inhibitory effects of the enteric nervous system, leading to an increase in gastric contractility and improved gastric emptying [5].

The safety and long-term efficacy of GES remain to be fully established. However, studies have reported relatively low rates of complications associated with GES placement, including infection, lead migration, and device malfunction. Long-term follow-up studies have shown that GES provides durable symptom relief and improved gastric emptying in many patients [5].

Other alternative treatments for refractory gastroparesis include enteral feeding, gastric bypass surgery, and botulinum toxin injections, but the evidence for these therapies needs to be more extensive and consistent. Enteral feeding is associated with significant complications and may be challenging to tolerate in some patients. Gastric bypass surgery may be effective in selected patients but is associated with a high risk of complications and morbidity. Botulinum toxin injections may improve symptoms in some patients, but the effects are typically short-lived, and the optimal dosing and injection sites are not well established [6].

In conclusion, GES is a promising treatment modality for refractory gastroparesis, offering significant improvements in symptom control, gastric emptying, and quality of life. The procedure is relatively safe and provides durable symptom relief for many patients. However, further research is needed to optimize patient selection, technique, and long-term outcomes. GES should be considered as a treatment option for patients with refractory gastroparesis who have failed conventional therapies, and its use should be individualized based on the patient's clinical presentation and preferences.

## Conclusions

This case report highlights the potential benefits of gastric electrical stimulation as a treatment option for refractory gastroparesis. Our patient, who had failed to achieve adequate symptom control with conventional therapies, significantly improved her gastroparesis symptoms following the placement of a gastric electrical stimulator. Further studies are needed to establish this treatment modality's long-term efficacy and safety.
